# The immune cell landscape of glioblastoma patients highlights a myeloid-enriched and immune suppressed microenvironment compared to metastatic brain tumors

**DOI:** 10.3389/fimmu.2023.1236824

**Published:** 2023-10-23

**Authors:** Beatrice Musca, Maria Giovanna Russo, Ada Tushe, Sara Magri, Greta Battaggia, Laura Pinton, Camilla Bonaudo, Alessandro Della Puppa, Susanna Mandruzzato

**Affiliations:** ^1^Immunology and Molecular Oncology, Veneto Institute of Oncology IOV – IRCCS, Padova, Italy; ^2^Department of Surgery, Oncology and Gastroenterology, University of Padova, Padova, Italy; ^3^Neurosurgery, Department of NEUROFARBA, University Hospital of Careggi, University of Florence, Florence, Italy

**Keywords:** brain metastases, glioblastoma, tumor microenvironment, tumor-associated macrophages, myeloid cells

## Abstract

**Introduction:**

Brain metastases (BrM), which commonly arise in patients with melanoma, breast cancer and lung cancer, are associated with a poor clinical prognosis. In this context, the tumor microenvironment (TME) plays an important role since it either promotes or inhibits tumor progression. Our previous studies have characterized the immunosuppressive microenvironment of glioblastoma (GBM). The aim of this study is to compare the immune profiles of BrM and GBM in order to identify potential differences that may be exploited in their differential treatment.

**Methods:**

Tumor and/or blood samples were taken from 20 BrM patients and 19 GBM patients. Multi-parametric flow cytometry was used to evaluate myeloid and lymphoid cells, as well as the expression of immune checkpoints in the TME and blood. In selected cases, the immunosuppressive ability of sorted myeloid cells was tested, and the *ex vivo* proliferation of myeloid, lymphoid and tumor cell populations was analyzed.

**Results:**

High frequencies of myeloid cells dominated both the BrM and GBM landscapes, but a higher presence of tumor-associated macrophages was observed in GBM, while BrM were characterized by a significant presence of tumor-infiltrating lymphocytes. Exhaustion markers were highly expressed in all T cells from both primary and metastatic brain tumors. *Ex vivo* analysis of the cell cycle of a single sample of a BrM and of a GBM revealed subsets of proliferating tumor cells and blood-derived macrophages, but quiescent resident microglial cells and few proliferating lymphocytes. Macrophages sorted from a single lung BrM exhibited a strong immunosuppressive activity, as previously shown for primary GBM. Finally, a significant expansion of some myeloid cell subsets was observed in the blood of both GBM and BrM patients.

**Discussion:**

Our results define the main characteristics of the immune profile of BrM and GBM, which are distinguished by different levels of immunosuppressive myeloid cells and lymphocytes devoid of effector function. Understanding the role of the different cells in establishing the metastatic setting is critical for improving the therapeutic efficacy of new targeted immunotherapy strategies.

## Introduction

Brain metastases (BrM) are the most frequent intracranial tumors: the incidence of newly diagnosed BrM is three to ten times that of newly diagnosed primary malignant brain tumors, considered between 9% and 17% according to various studies, although the exact percentage is thought to be higher (1–3). It is hypothesized that this incidence is increasing due to improved cancer survival, an aging of the population, increased awareness of the disease and better diagnostic tests. Lung cancer is considered the most common source of BrM (39-56%), followed by breast cancer (13-30%), melanoma (8-11%), renal cell cancer (2-6%) and colorectal cancer (6-9%). However, the primary tumor may also be unknown in 2-14% of cases. Metastatic breast cancer predominates in women, whereas lung cancer is the most frequent source of BrM in the male population (1–4).

At the onset of neurological symptoms, 50-70% of BrM appear on an MRI scan, and while lung cancer is the most frequent source of brain metastases, melanoma has the highest propensity of all systemic malignant tumors to metastasize to the brain (5, 6). Surgery is the preferred treatment for BrM, especially for single lesions, followed by radiation therapy; these treatments are frequently combined. BrM are generally well demarcated from the surrounding brain parenchyma, although infiltrative growth patterns have been observed and described (7). Sodium fluorescein- or 5-aminolevulinic acid (5-ALA)-induced fluorescence may be used to improve the surgical strategy of tumor removal (8–10) and maximize the extent of resection, especially in eloquent areas (11).

The onset of the metastatic setting depends on the establishment of a continuous interplay between cancer cells and the surrounding tumor microenvironment (TME) (12–14). In this process, the main source of variability among metastases of different origins is at the level of the immune infiltrate that may be shaped by the cancer type (15, 16), although lymphocytes and macrophages remain the most representative populations (15, 17). In this respect, T cells are characterized by a variety of activation states (17–19), a high expression of both co-stimulatory and co-inhibitory receptors (15) and a different spatial distribution in the TME (18, 20). As far as glioblastoma is concerned, two subsets of macrophages are present in the brain parenchyma, i.e. brain-resident microglial cells (MG) and bone marrow-derived macrophages (BMDM). These populations display different antigen-presenting properties (15–17), and transcriptional and polarization profiles (21), thus helping cancer cells to colonize the brain and assisting them in all the processes of the metastatic cascade (12, 22).

Several studies have shown the diversity of human BrM and glioma TME, in terms of both cell frequency (15, 16) and spatial distribution of cells in the brain parenchyma (23). We have also extensively studied the TME of gliomas and compared the immune composition with increased tumor grading (24), showing that the immune cell infiltrate of glioblastoma (GBM), is characterized by a high proportion of BMDM (24) and a paucity of T cells with the morphology and markers of exhausted cells (25). In addition, we investigated the immune suppressive activity of BMDM toward T cell proliferation in gliomas, and found that it increases from the marginal to the central region of the tumor (24) but, to the best of our knowledge, this information for BrM is, to some extent, conflicting and limited to mouse models. In fact, while a study in a breast-to-brain mouse model showed the ability of BMDM to inhibit T cell activation in the expression of CD69, IFNγ and Granzyme B (26), another one demonstrated that CNS-resident microglial cells are responsible for promoting the immune suppressive microenvironment in BrM (27). However, no information regarding the immune suppressive potential of myeloid cells in the TME of human BrM is present.

Additionally, it has to be considered that the presence of a tumor in the brain may trigger a response in the peripheral blood. Therefore, the analysis of the immune populations to the peripheral blood may be a source of relevant information (28). However, while the majority of the studies on BrM are focused on the tumor, the information regarding blood immune populations is infrequent.

Therefore, given the importance of the immune cell composition in the TME and the systemic responses induced by the presence of tumors in the brain, in this study, we compare the immune landscape of GBM with that of the most prevalent BrM both at the tumor and blood level, with a particular focus on myeloid cells and their immune suppressive activity in the human metastatic setting.

## Methods

### Patient characteristics

BrM patients in this study were consecutively recruited at the Department of Neurosurgery in Padova (from 2016 to 2018) and Florence University Hospitals (from 2021 to 2022) in Italy. GBM patients were recruited in Padova between 2016 and 2017 and in Florence in 2021. All participant characteristics are detailed in **Table 1**. Briefly, 19 first-surgery GBM patients and 20 BrM patients were enrolled, and freshly resected tumor samples and peripheral blood were collected. Thirteen and 19 tumor specimens were obtained from BrM and GBM patients, respectively, together with 17 and 16 blood samples drawn immediately before induction of anesthesia. In addition, peripheral blood was collected from 18 healthy donors (HD).

**Table 1:** Participants’ characteristics.

	BrM		GBM		HD
	Blood	Tumor	Blood	Tumor	Blood
**Total *n* of patients**	17	13	16	19	18
Sex					
***Male, n***	7	6	10	12	13
***Female, n***	10	7	6	7	5
**Median age**	57	61	67	67	61
***Range***	29-78	43-78	46-80	46-80	36-84
Primary tumor					
***Breast***	4	3	N/A	N/A	N/A
***Lung***	6	3	N/A	N/A	N/A
***Bladder***	1	1	N/A	N/A	N/A
***Ovary***	1	1	N/A	N/A	N/A
***Melanoma***	5	5	N/A	N/A	N/A

N/A, not applicable.

All the experiments were approved by the ethics committees of the Veneto Institute of Oncology–IRCCS of Padova, Italy (MDSC_SNC 2016/13) and the Padova and Florence University Hospitals (NOI_NCH 1536/19). All patients gave their written informed consent, and the study was conducted in accordance with the Declaration of Helsinki.

### Tumor specimen processing

Immediately after resection, tumor specimens were immediately transferred to MACS^®^ Tissue Storage Solution (Miltenyi Biotec, Bergisch Gladbach, Germany) and stored at 4°C until processing. Sample digestion was performed as reported previously (24). Briefly, samples were washed with 0.9% sodium chloride solution to remove blood traces and mechanically and enzymatically digested using Human Tumor Dissociation Kit (Miltenyi Biotec) and the gentleMACS™ Octo Dissociator (Miltenyi Biotec), following manufacturer’s instructions for soft tumor digestion. For the cell sorting experiment, the single-cell suspension was subjected to immunomagnetic bead-based separation using human CD33 MicroBeads (Miltenyi Biotec) according to the manufacturer’s instructions, to isolate CD33^high^ cells.

### Phenotypic analysis

Fresh peripheral blood samples were stained with fluorescently-labeled monoclonal antibodies (mAb) as described in (29) to identify different circulating myeloid populations. In detail, four distinct subsets of myeloid-derived suppressor cells (MDSC), polymorphonuclear cells (PMN) and monocytes were identified according to the following antibody panel: anti-CD11b Alexa Fluor 700 (BD Biosciences, Becton Dickinson, Franklin Lakes, NJ, USA), anti-CD14 APC-H7 (BD Biosciences), anti-CD15 V450 (BD Biosciences), anti-CD33 PE-Cy7 (eBioscience, Thermo Fisher Scientific, Waltham, MA, USA), anti-IL4Rα PE (R&D Systems, Minneapolis, MN, USA), anti-lineage cocktail 1 (Lin 1) FITC (BD Biosciences) and anti-HLA-DR APC (BD Biosciences). Fluorescence minus one (FMO) negative controls were prepared for HLA-DR and IL4Rα. The overall staining procedure was standardized as reported previously (29).

Tumor specimens were stained with different antibody cocktails for the analysis of the immune infiltrate. To this end, single-cell suspensions obtained from freshly resected tumors were stained with LIVE/DEAD™ Fixable Aqua (Life Technologies, Thermo Fisher Scientific), anti-CD45 BV421 (BD Biosciences), anti-CD33 PE-Cy7 (eBioscience, Thermo Fisher Scientific) or anti-CD33 APC (BD Biosciences), anti-HLA-DR APC (BD Biosciences), anti-CD49d PE (BioLegend, San Diego, CA, USA), anti-CD3 PE-Cy7 (Beckman Coulter, Indianapolis, Indiana, USA) or anti-CD3 APC-H7 (BD Biosciences), anti-CD8 APC-H7 (BD Biosciences), anti-CD4 BV785 (BioLegend), anti-lymphocyte-activation gene 3 (LAG-3) FITC (AdipoGen, San Diego, CA, USA), anti-programmed cell death protein 1 (PD-1) PE (Miltenyi Biotec) and anti-T cell immunoreceptor with Ig and ITIM domains (Tigit) PE-Cy7 (BioLegend). FMO tubes were prepared as negative controls for PD-1, LAG-3 and Tigit.

Multicolor flow cytometry analysis was performed on an LSRII flow cytometer (BD Biosciences), and data analysis was carried out with FlowJo™ software (BD Biosciences).

### Immunosuppressive activity assay

The immune suppressive activity of CD33^high^ myeloid cells sorted from a lung BrM was evaluated by determining the proliferation of allogenic CellTrace™-labeled peripheral blood mononuclear cells (PBMC) isolated from HD buffy coats as described previously (24). Briefly, PBMC, stained with 0.5 μM CellTrace™ Violet Cell Proliferation Kit (Invitrogen, Molecular Probes, MA, USA), according to the manufacturer’s instructions, were activated with coated 1 μg/mL anti-CD3 and 5 μg/mL soluble anti-CD28 (BioLegend) mAb and co-cultured in flat-bottom 96-well plates at 1:1 ratio with CD33high cells sorted from a lung brain metastasis. After four days at 37°C, 5% CO_2_, T cell proliferation was assessed by staining cells with anti-CD3 PE-Cy7 mAb (Beckman Coulter) for flow cytometry analysis and analyzed with BD™ LSRII flow cytometer (BD Biosciences). T cell proliferation was determined by calculating the absolute number of proliferating T cells using Trucount™ tubes (BD Biosciences). All data were normalized by assuming that the proliferation of T cells alone was 100%.

### Evaluation of cell subset proliferation by BrdU incorporation

Single-cell tumor suspensions were stained with the BD Pharmingen™ FITC BrdU Flow Kit (BD Biosciences) according to the manufacturer’s instructions in order to characterize the cell cycle. Briefly, cells were incubated with bromodeoxyuridine (BrdU) for one hour and, after incubation, stained with LIVE/DEAD™ Fixable Aqua (Life Technologies, Thermo Fisher Scientific), anti-CD45 BV421 (BD Biosciences), anti-HLA-DR APC (BD Biosciences) and anti-CD49d PE (BioLegend), as described in the “Phenotypic analysis” paragraph. Cells were then fixed and permeabilized with BD Cytofix/Cytoperm Buffer and BD Cytoperm Permeabilization Buffer Plus, both provided in the kit, and treated with DNase to expose the incorporated BrdU. At the end, cells were stained with an anti-BrdU FITC mAb, to evaluate its incorporation, and with 7-aminoactinomycin D (7-AAD), to stain the total DNA content for cell cycle analysis. The LSRII flow cytometer (BD Biosciences) was used to acquire data and the FlowJo™ software (BD Biosciences) was used for the analysis.

### Statistical analysis

SigmaPlot software (Systat Software Inc., San Jose, CA, USA) was used for data statistical analysis. The Mann-Whitney test and Student’s *t*-test were performed, with *P* values < 0.05 considered statistically significant. All tests were two-sided, and for the sake of brevity, the lack of significance was not reported. Pearson’s correlation was performed to assess the correlation between different parameters.

## Results

### Analysis of the myeloid infiltrating cells in the tumor microenvironment of BrM and primary GBM

**Figure 1:**
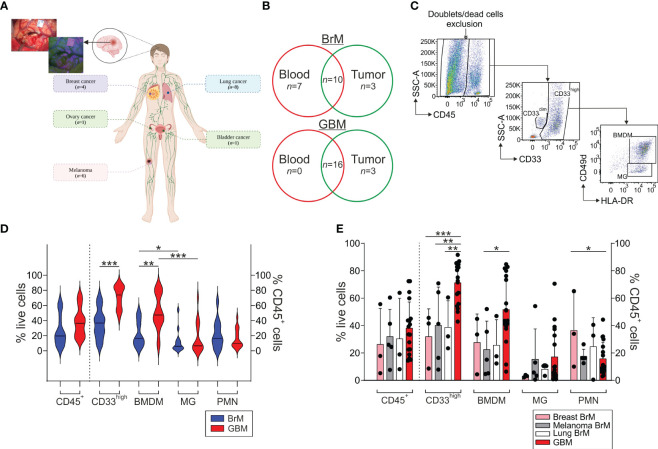
Analysis of the myeloid infiltrate in the TME of BrM and GBM. **(A)** Schematic representation of the localization of primary BrM tumors analyzed in the study. In the upper left, a representative surgical view under white and blue light after 5-ALA administration to a BrM patient. Figure created with biorender.com. **(B)** Venn diagram of the samples included in the study. Venn diagram showing BrM (upper part) and GBM (lower part) blood and tumor samples included in the analysis. Overlapping numbers in the graphs refer to the numbers of patients for which matched tumor and blood samples were obtained. **(C)** Representative flow cytometry gating strategy for the identification of the different myeloid subsets in tumor samples. After morphological evaluation and the exclusion of doublets and dead cells, leukocytes were identified on the basis of their CD45 expression. Macrophages and PMN were further discriminated in the CD45^+^ gate based on their differential CD33 expression, with PMN identified as CD33^dim^ cells and macrophages as CD33^high^ cells. Finally, BMDM and MG were further differentiated in the CD33^high^ subset either based on the combined expression of CD49d and HLA-DR or CD33 and HLA-DR, with BMDM distinguished as CD49d^+^/HLA-DR^+^ or CD33^high^/HLA-DR^+^ and MG as CD49d^-^/HLA-DR^+^ or CD33^high^/HLA-DR^dim^, respectively. **(D)** Analysis of the frequency of the leukocyte subsets in the TME of BrM and GBM. CD45^+^ cells were calculated among live cells, while CD33^high^, BMDM, MG and PMN were gated among CD45^+^ leukocytes. Violin plots show the frequency distribution and median of tumor-infiltrating leukocytes in the whole BrM (blue plots) and GBM (red plots) (n = 14 for BrM, except for PMN [n = 13]; n = 18 for GBM). Comparison by Mann-Whitney test. * <.05; ** <.01; *** ≤.001. **(E)** Analysis of the leukocyte infiltrate in the TME of BrM according to their primary tumor site. Bars show the mean ± standard error (SE) of the frequency of tumor-infiltrating leukocytes in BrM from breast cancer (pink bars, n = 3), melanoma (grey bars, n = 5), lung cancer (white bars, n = 3) and GBM (red bars, n = 18). Each black dot represents a sample. Comparison by t-test. * <.05; ** <.01; *** ≤.001.

Twenty patients with BrM and 19 patients with primary GBM undergoing their first surgery were enrolled in this study. BrM derived from different primary tumors, including melanoma (*n* = 6), lung (*n* = 8), bladder (*n* = 1), ovary (*n* = 1) and breast cancer (*n* = 4) (**Figure 1A**). When 5-ALA-assisted surgery was used for tumor resection, all tissue specimens collected were derived from the bright PpIX fluorescent area; in all the other cases, specimens were obtained from the central tumor region. All of the participants’ characteristics are summarized in **Table 1** and **Figure 1B**.

Using multi-parametric flow cytometry, we investigated the myeloid and T cell infiltrate present in the TME; an example of the gating strategy used is shown in **Figure 1C**. Both BrM and GBM had a broad CD45^+^ leukocyte infiltrate, with a range between 5.3% and 63.9% among live cells (median 19.6%) for BrM and 14.4% and 72.9% for GBM (median 36.4%), as shown in **Figure 1D**. In both cases, the bulk of the leukocyte infiltrate was composed of CD33^high^ macrophages, with a higher proportion in GBM (median 36.6% vs. 73.85% in BrM and GBM, respectively, *P* ≤ 0.001). We then dissected the contribution of resident vs. blood-derived macrophages in the CD33^high^ cells as reported previously (24) and identified BMDM as CD45^+^/CD33^high^/HLA-DR^+^/CD49d^+^ cells and microglia (MG) as CD45^+^/CD33^high^/HLA-DR^+^/CD49d^-^ (**Figure 1C**), and observed a higher frequency of BMDM in GBM compared to BrM (**Figure 1D**, median 16.3% in BrM vs. 47.3% in GBM, *P* = 0.003). As previously reported, a significantly higher presence of BMDM compared to MG was observed in GBM, and the same also held true for BrM, although the presence of BMDM was significantly higher in GBM than BrM.

We then analyzed the myeloid infiltrate of BrM according to their primary site for breast (*n* = 3), melanoma (*n* = 5) and lung BrM (*n* = 3) and compared them to GBM. We found no significant difference in terms of the frequency of leukocytes, but a trend toward an increase of CD45^+^ cells in GBM (**Figure 1E**). Instead, significant differences emerged by analyzing the myeloid populations, with a higher presence of CD33^high^ cells in GBM compared to breast, melanoma and lung BrM. Regarding BMDM, their presence was significantly higher in GBM compared to melanoma BrM (**Figure 1E**, *P* = 0.0222). No significant differences were observed for the presence of PMN, with the exception of breast BrM, in which we found a higher presence compared to GBM (**Figure 1E**, *P* = 0.0343).

Overall, these results suggest that BrM are characterized by a consistent myeloid infiltrate, mainly composed of BMDM and PMN, with a pattern similar to that of GBM, albeit at a lower level.

### Analysis of the T cell infiltrate and the expression of exhaustion markers

Together with the myeloid populations, we also analyzed the presence of T cells in the immune infiltrate, which is an important component of the TME of BrM (15–17), given their potential role as antitumor effectors. A representative gating strategy for the analysis of lymphoid populations is reported in **Supplementary Figure 1** of the **Supplementary Material** section.

**Figure 2:**
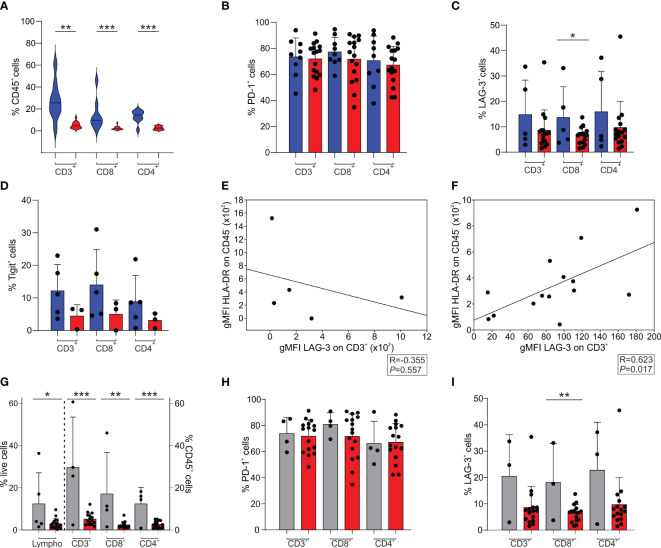
T cell infiltrate and exhaustion markers in the TME of BrM and GBM. **(A-D)** CD3^+^, CD8^+^ and CD4^+^ cells were selected among CD45^+^ leukocytes. CD8^+^ cells were gated as CD3^+^/CD8^+^ or CD3^+^/CD4^-^ cells, while CD4^+^ cells were identified as CD3^+^/CD8^-^ or CD3^+^/CD4^+^ cells. Blue and red plots refer to BrM and GBM, respectively. **(A)** Analysis of the frequency of T cell subsets in the TME of the entire BrM and GBM. Violin plots illustrate the distribution and the median of the frequency of tumor-infiltrating leukocytes in the entire BrM and GBM (n = 9 for BrM; n = 16 for GBM). Comparison by Mann-Whitney test. ** <.01; *** <.001. **(B)** Percentage of PD-1^+^ cells in the total CD3^+^ population and in the CD8^+^ and CD4^+^ T cell subsets (n = 9 for BrM; n = 16 for GBM). Each dot represents a sample. **(C)** Percentage of LAG-3^+^ cells in the total CD3^+^ population and in the CD8^+^ and CD4^+^ T cell subsets (n = 5 for BrM; n = 16 for GBM). Each dot represents a sample. Comparison by t-test. * <.05. **(D)** Percentage of Tigit^+^ cells in the total CD3^+^ population and in the CD8^+^ and CD4^+^ T cell subsets (n = 5 for BrM; n = 3 for GBM). Each dot represents a sample. **(E, F)** Correlation between LAG-3 expression (expressed as geometric mean fluorescence intensity, gMFI) on CD3^+^ cells and HLA-DR expression on CD45^-^ cells (i.e., tumor cells) in BrM **(E)** and GBM **(F)**. Pearson correlation on 5 **(E)** and 14 **(F)** paired samples. **(G-I)** T cell infiltrate and exhaustion markers in the TME of melanoma BrM and GBM. Lymphocytes were identified using morphological parameters in the CD45^+^ cell fraction. CD3^+^, CD8^+^ and CD4^+^ cells were gated among CD45^+^ leukocytes. CD8^+^ cells were selected as CD3^+^/CD8^+^ or CD3^+^/CD4^-^ cells, while CD4^+^ cells were identified as CD3^+^/CD8^-^ or CD3^+^/CD4^+^ cells. Grey and red plots refer to melanoma BrM and GBM, respectively. **(G)** Analysis of the frequency of lymphocyte subsets in the TME of BrM from melanoma and GBM. Bars show the mean ± SE of the frequency of tumor-infiltrating leukocytes in BrM from melanoma and GBM (n = 5 for melanoma BrM and n = 18 for GBM for total lymphocytes; n = 4 for melanoma BrM and n = 15 for GBM for CD3^+^, CD8^+^ and CD4^+^ subsets). Each dot represents a sample. Comparison by t-test. * <.05; ** <.01; *** ≤.001. **(H)** Percentage of PD-1^+^ cells in the total CD3^+^ population and in the single CD8^+^ and CD4^+^ subsets (n = 4 for BrM; n = 16 for GBM). Each dot represents a sample. **(I)** Percentage of LAG-3^+^ cells in the total CD3^+^ population and in the single CD8^+^ and CD4^+^ subsets (n = 3 for BrM; n = 16 for GBM). Each dot represents a sample. Comparison by t-test. ** ≤.01.

Compared to GBM, we observed a significant increase in the presence of tumor-infiltrating CD3^+^ T cells in BrM both in the CD8^+^ and in the CD4^+^ subsets (**Figure 2A**). We then analyzed the expression of the PD-1 (**Figure 2B**), LAG-3 (**Figure 2C**) and Tigit (**Figure 2D**) immune checkpoints and compared it to that of T cells present in the GBM microenvironment. A high frequency of PD-1^+^ T cells was present in the TME of both BrM and GBM, without significant differences (**Figure 2B**). The percentage of LAG-3^+^ (**Figure 2C**) and Tigit^+^ (**Figure 2D**) cells was lower than that of PD-1^+^ T cells, with a tendency for a higher presence in BrM, suggesting a lack of T cell functional response in these tumors. As LAG-3 binds to HLA class II, we analyzed a possible role of tumor cells in the immune suppressive TME, and interestingly, found that the expression of LAG-3 and that of HLA-DR are significantly correlated in GBM (**Figure 2F**). Instead, no significant correlation was found in BrM (**Figure 2E**), suggesting an immune suppressive role for GBM tumor cells, but not for BrM.

When we independently analyzed melanoma BrM (*n* = 4), which have a peculiar immune infiltrate, as these tumors are considered hot tumors, with a sustained lymphocyte infiltrate, and a high number of point mutations, we observed a significantly higher infiltration of lymphocytes, evaluated by morphological SSC-A parameters combined with CD45 expression, and by the T cell markers CD3, CD4 and CD8 (**Figure 2G**). In terms of immune checkpoint expression, PD-1 expression on T cells was high in both melanoma BrM and GBM (**Figure 2H**), while LAG-3 expression was reduced (**Figure 2I**), as previously mentioned (**Figure 2C**).

### Analysis of the myeloid populations in the peripheral blood of BrM and GBM patients

It has been clearly demonstrated by us and by others that cancer patients have an altered myelopoiesis (30) and that an expansion of MDSC is found in glioma patients, the level of which is a component of a prognostic model for GBM patients (28). We thus analyzed the levels of several myeloid populations, including four different MDSC subsets, monocytes (evaluated as CD14^+^ cells) and PMN (evaluated as CD15^+^ cells), in the peripheral blood of BrM and GBM patients and compared them with a group of HD matched for sex and age. The four MDSC subsets were identified as CD14^+^/IL4Rα^+^ (MDSC1), CD15^+^/IL4Rα^+^ (MDSC2), Lin^−^/HLA-DR^−^/CD11b^+^/CD33^+^ (MDSC3) and CD14^+^/HLA-DR^−^ (MDSC4), as previously described (31).

**Figure 3:**
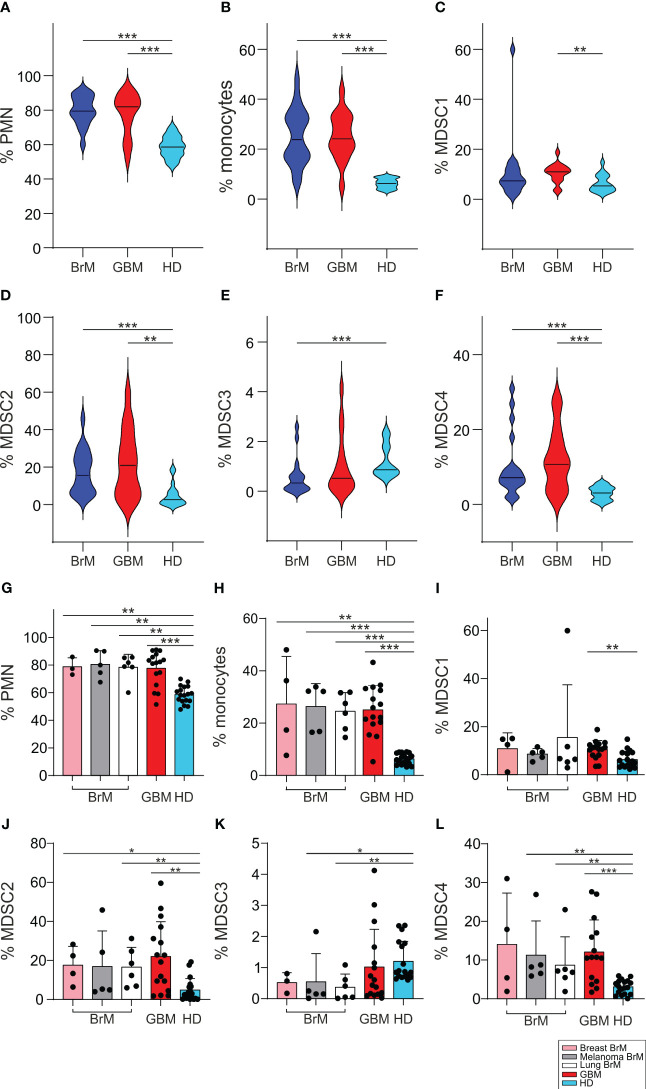
Analysis of circulating myeloid populations in the peripheral blood of BrM and GBM patients. **(A-F)** PMN, monocytes and MDSC1-4 were evaluated in the peripheral blood of BrM (blue plots) and GBM (red plots) and compared to a set of HD (light blue plots). Violin plots illustrate the distribution and the median of the frequency of **(A)** PMN (n = 18 for BrM; n = 16 for GBM; n = 18 for HD), **(B)** monocytes (n = 19 for BrM; n = 16 for GBM; n = 18 for HD), **(C)** MDSC1 (n = 19 for BrM; n = 16 for GBM; n = 18 for HD), **(D)** MDSC2 (n = 19 for BrM; n = 16 for GBM; n = 18 for HD), **(E)** MDSC3 (n = 18 for BrM; n = 16 for GBM; n = 18 for HD) and **(F)** MDSC4 (n = 19 for BrM; n = 15 for GBM; n = 18 for HD) in the peripheral blood of BrM and GBM patients. Comparison by Mann-Whitney test. ** <.01; *** ≤.001. **(G-L)** Analysis of circulating myeloid cells in the peripheral blood of BrM separated according to their primary site. Bars show the mean ± SE of the frequency of PMN **(G)**, monocytes **(H)** and MDSC1-4 **(I-L)** in BrM from breast cancer (pink bars), melanoma (grey bars), lung cancer (white bars), GBM (red bars) and in a set of HD (light blue bars) (n = 4 for breast BrM, except for PMN and MDSC3 [n = 3], n = 5 for melanoma BrM, n = 6 for lung BrM, n = 16 for GBM, except for PMN [n = 15], and n = 18 for HD). Each dot represents a sample. Comparison by Mann-Whitney test. * <.05; ** <.01; *** ≤.001.

As shown in **Figures 3A–F**, we found that the presence of both primary and metastatic brain tumors was associated with elevated levels of circulating myeloid cells, including PMN, monocytes and MDSC1, 2 and 4 compared to HD. In contrast, a significant drop in the level of MDSC3 was observed in BrM. However, there were no remarkable differences between BrM and GBM for any of the myeloid subsets.

When we separated BrM on the basis of primary tumors, from breast cancer (*n* = 4), melanoma (*n* = 5) and lung cancer (*n* = 6), we found no significant differences between BrM from different sites or between them and GBM (**Figures 3G-L**).

The expansion of MDSC cell subsets 1-4 has already been observed in melanoma (29), in meningioma (32), in pancreatic cancer (33) and in glioma patients (28), and thus these data indicate that a sustained tumor-induced myelopoiesis is also present in metastatic patients. In addition, since we showed that some of these MDSC subsets may have diagnostic and prognostic value in glioma patients (28), these results highlight their potential role as biomarkers also in brain metastases.

### Macrophages from a lung BrM are endowed with immune suppressive potential

Previous studies from our laboratory demonstrated that macrophages from GBM samples possess an immune suppressive activity that depends not only on their ontogeny but also on the tumor context, since their immunosuppressive potential increases as they migrate to the center of the lesion (24). Therefore, we sought to determine whether macrophages also exert an immune suppressive role in BrM and had the opportunity to perform this analysis only from a single case of a lung metastasis.

**Figure 4:**
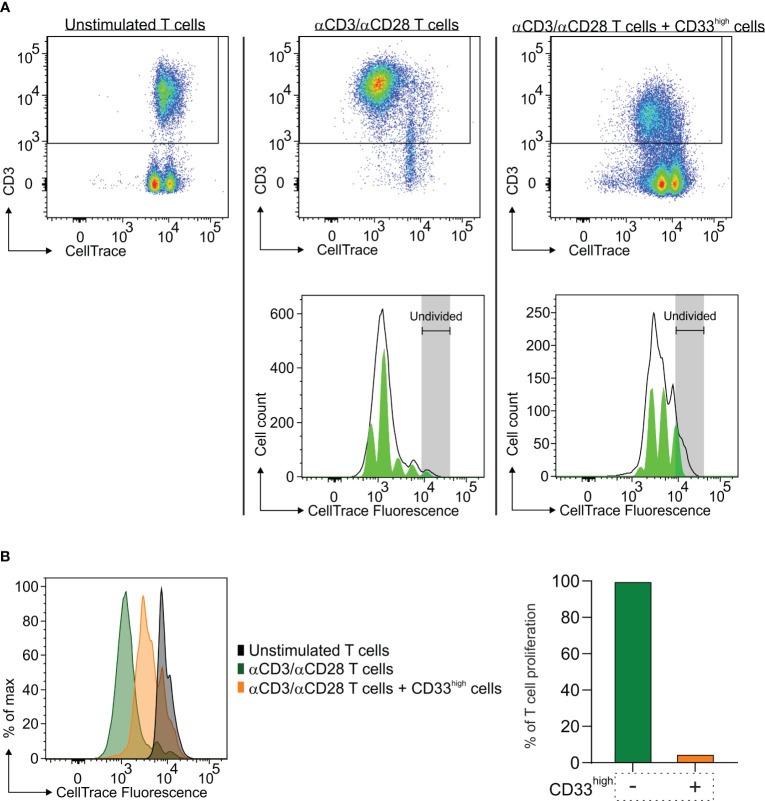
Immune suppressive activity of macrophages in the TME of a lung BrM. CD33^high^ cells were isolated from a single-cell suspension of a lung BrM using anti-CD33 immunomagnetic beads and cultured for four days with αCD3 and αCD28 mAb-stimulated PBMC in a 1:1 ratio. **(A)** Dot plots show the proliferation of unstimulated T cells (left), of αCD3/αCD28 mAb-stimulated T cells (middle) and of T cells cultured in the presence of CD33^high^ cells (right). Green histograms below represent the model of the proliferation of T cells in the corresponding conditions, with each peak corresponding to a proliferation generation. **(B)** On the left, CellTrace histograms of unstimulated T cells alone (black plot), αCD3/αCD28 mAb-stimulated T cells (green plot) and T cells in the presence of sorted CD33^high^ cells (orange plot). On the right, the bars represent the value of quantitative T cell proliferation normalized assuming the proliferation of T cells alone (green bar) to be 100%.

To that end, after enriching CD33^high^ cells to 88% of live cells (**Supplementary Figure 2**), we tested their immune suppressive potential toward the proliferation of activated T cells. As shown in **Figure 4**, macrophages exerted a strong suppression on T cell proliferation, as evidenced by the down-regulation of CD3 expression on T cells (**Figure 4A**), but also by the higher CellTrace™ fluorescence intensity (**Figure 4B**, left part) and the quantitative reduction in T cell proliferation (**Figure 4B**, right part).

### Evaluation of cell subset proliferation in the brain TME

**Figure 5:**
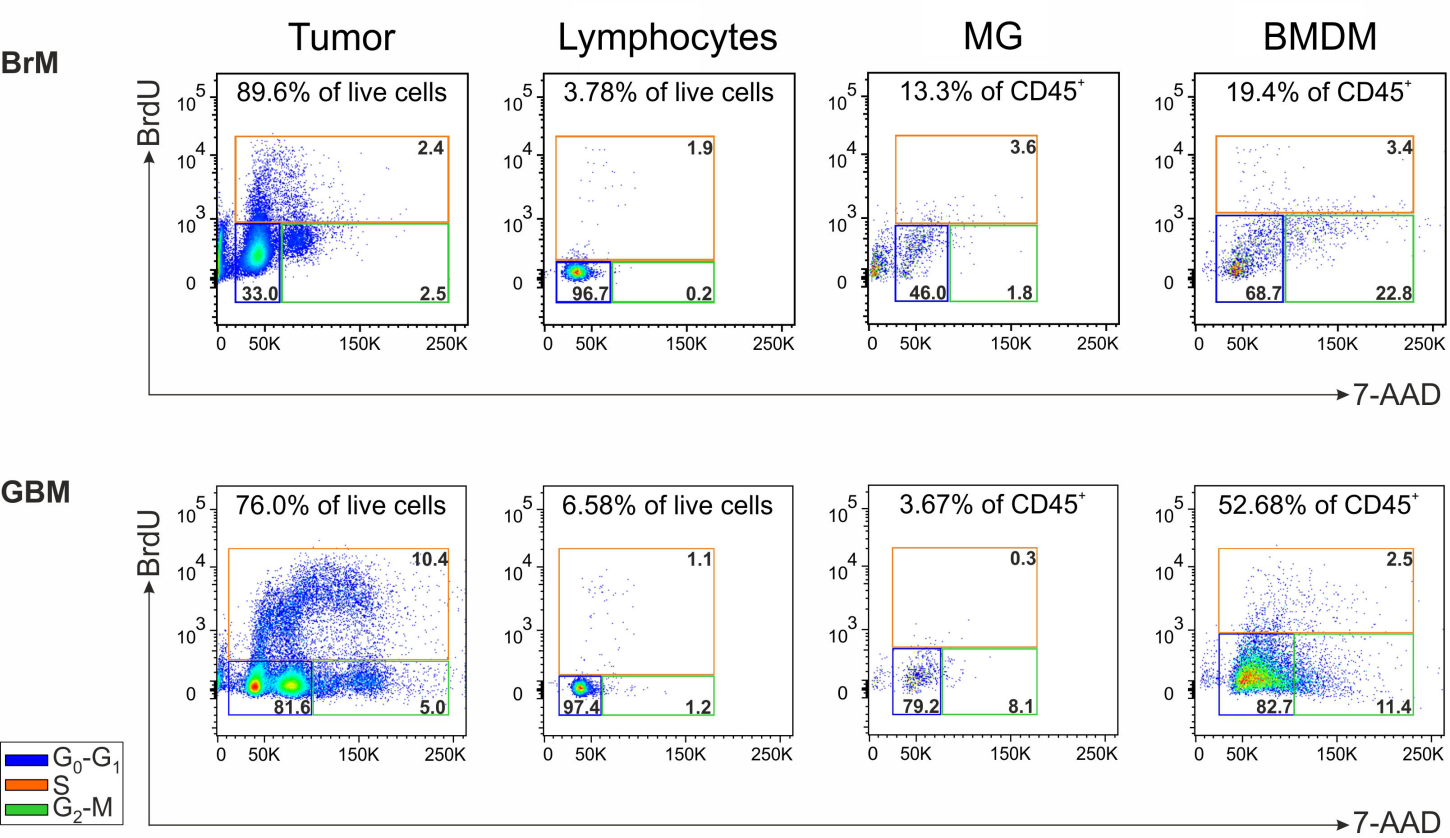
Analysis of the cell proliferation in the TME of a lung BrM and a GBM. Cell cycle analysis of the main cell subsets present in the TME of a representative lung BrM (upper section) and GBM (lower section). Cell suspensions from dissociated specimens were pulse-labeled with BrdU for 1 hour and then counterstained with 7-AAD for flow cytometry analysis. For each subset, the different cell cycle phases were discriminated by plotting BrdU vs. 7-AAD and color-coding them as follows: blue square for G_0_-G_1_; green square for G_2_-M; orange square for S. The numbers in the upper part of each box refer to the percentage of each cell subset in the tumor specimen, while the numbers inside the colored boxes indicate the percentage of cells in the corresponding cell cycle phase.

We sought to determine if cell subsets present in the TME maintain the ability to proliferate *ex vivo*, immediately after resection and without any additional stimulation. To achieve this, BrdU incorporation was conducted in cell suspensions from a single GBM and a single lung metastasis, and the cell cycle was analyzed after 1 hour in both BMDM and MG macrophages, lymphocytes and tumor cells (evaluated as CD45^-^ cells) **Supplementary Figure 3**. As expected, the main cell subset that entered the active phases of the cell cycle (i.e., the S and G_2_-M phases) was represented by tumor cells, particularly in the GBM, in which two distinct cell populations with a different DNA content could be detected in both G_0_-G_1_ and G_2_-M phases. In addition, in the GBM we observed that the proliferation of lymphocytes and MG cells was low or absent, although 1.1% of the lymphocytes incorporated BrdU suggesting an active S phase. Notably, BMDM exhibited a cell subset corresponding to 1.3% of CD45^+^ infiltrating leukocytes entering the S phase (i.e., 2.5% of BMDM in the S phase x 52.68% of BMDM in CD45^+^ cells) (**Figure 5**). Similarly, proliferating BMDM were present also in the BrM, although to a lower extent. Although these data were obtained from two cases, and thus needs to be confirmed on a larger cohort, to the best of our knowledge, they represent the first evidence suggesting that BMDM proliferate in the TME; these data also reinforce the notion that blood-derived and resident macrophages differ not only in terms of immune suppressive activity but also in terms of proliferative ability.

## Discussion

Cancer cells that metastasize to the brain need to adapt to a very peculiar microenvironment that is radically different from the site of origin. The colonization of the tumor cells at a distant site also includes the induction of immune evasion mechanisms in the TME, such as the infiltration of immune cells with tumor-promoting activity. Our previous studies on primary brain tumors identified a typical immune landscape mainly composed of immune suppressive macrophages both in meningiomas and in GBM (24, 32). Another recurrent characteristic of the TME in primary brain tumors is the scarcity of lymphocytes, especially in GBM, which is a feature that defines a typical cold microenvironment. Recently, Wischnewski and collaborators shed light on the composition and potential role of T cells present in primary and metastatic brain tumors, and identified by a transcriptomic approach a subgroup of patients with brain metastases characterized by potential tumor-reactive T cells that were clonally expanded (19). However, cells with such characteristics were not found in glioma patients, emphasizing once again the signs of a cold tumor microenvironment in which anti-tumor T cells do not have the possibility to exert their functional activity. Among the mechanisms fueling this condition, immune suppression exerted by BMDM plays a central role (24).

The findings in this study highlight that a sustained recruitment of blood-derived macrophages occurs even in the most common BrM and a preliminary result indicates that such cells possess immune suppressive ability (**Figure 4**), as previously seen in GBM (24). This supports the hypothesis that the recruitment of myeloid cells in the brain parenchyma of a growing tumor is not only a defining trait, but it also allows the tumor to avoid its immune-mediated destruction.

We found increased levels of different myeloid cell subsets in the blood of both BrM and GBM patients, compared to matched healthy donors, but no significant differences between the two types of tumors for any of the myeloid subsets, although BMDM had significantly higher levels in the TME of GBM compared to BrM. Levels of myeloid cells in the blood at surgery is a snapshot of a phenomenon that has probably been ongoing for years in the case of GBM at diagnosis, and for a variable length of time for metastatic brain tumors, and therefore a simple correlation between the two districts cannot be made. In addition, trafficking, proliferation, differentiation and survival of myeloid cells in cancer patients is a phenomenon that depends on several factors such as chemokines and their receptors, differentiation in the TME and rate of survival/death. The mechanism of monocyte differentiation in the TME is a concept that has been recently evaluated with single-cell transcriptomic studies in different human cancer types (34), and it revealed a complex phenotype of TAM and a monocyte reprogramming by the TME. In addition, these results suggest an intermediate state of monocytes migrating into tissues and differentiating into macrophages. Furthermore, the group of J. Joyce has demonstrated that the TAM transcriptomic changes in gliomas and in brain metastases are influenced not only by the brain TME, but also by the specific type of malignancy (16). In this work the greater changes in gene expression were observed in BMDM, compared to resident MG cells, indicating the high plasticity of these cells when colonizing a brain tumor. This finding points to recruited BMDM as cells that possess the ability to adapt to the new colonization site by exploiting a cell-specific program that depends on the tumor type. Another aspect that must be taken into account in this context is the possible role of cancer therapy in monocyte reprogramming which can affect monocyte progenitors and their differentiation, with a different outcome on their recruitment into the tumor (35).

Remarkably, the T cell infiltrate in the TME of BrM is significantly higher than that of GBM, suggesting that the priming of the antitumor T cell response is higher for tumors of extracranial origin, in line with the high mutational burden of melanoma and lung cancer. Other factors that could explain the higher frequency of T cells in BrM from extracranial tumors are the release of chemokines and the presence of molecular pathways expanding T cell trafficking and extravasation, like an increased expression of cellular adhesion molecules on endothelial cells (36).

However, T lymphocytes that are present in brain tumors show high levels of exhaustion markers, irrespective of the tumor’s origin. Thus, despite a higher presence of T cells in tumors of extracranial origin, it appears that their activity is hampered, as evidenced by the high expression of PD-1, LAG-3, and Tigit on their surface (**Figure 2**), and the concomitant immunosuppressive activity mediated by BMDM observed in a lung metastasis (**Figure 4**). The presence of immune checkpoints expressed at high levels on T cells points to their dysfunctional activity in both GBM and BrM. Large phase III studies with Nivolumab associated with chemo/radiotherapy did not improve survival in GBM patients, and it is expected that a single agent-based therapy will be insufficient to overcome GBM resistance. As far as it concerns BrM, the presence of inhibitory receptors should be analyzed in the context of each tumor type, along with all the known mechanisms of resistance, but in this case, there are clinical evidences that patients with brain metastases can benefit from ICI (immune checkpoint inhibitor) treatment, especially melanoma (37).

The presence of significant numbers of leukocytes in brain tumors also highlights the lack of a functioning blood-brain barrier (BBB), a fact that is also emphasized by the contrast enhancement pattern of these tumors in MRI (38). Although it is well known that the BBB is not homogeneously disrupted in brain tumors (39), the significant presence of leukocytes in these tumors argues in favor of the administration of drugs capable of activating an antitumor immune response. In line with this consideration, the presence of a TME with a strong immune suppressive trait should lead to the careful planning of immune interventions for brain tumors, where a combination of treatments that stimulate the immune system and block the TME’s main suppressive activities represents a new opportunity for the treatment of both primary and secondary tumors. To fully exploit this opportunity, a rational targeted approach that exploits the immune landscape and the functional and metabolic connections that sustain tumor growth is required. In this regard, we have recently demonstrated that by inhibiting heme oxygenase-1 (HO-1), a key metabolic enzyme of iron metabolism in BMDM, the immune suppressive activity of tolerogenic cells is alleviated and T cell proliferation is restored (40). However, while the activity of immune suppressive cells appears to be a valuable target for undermining tumor growth, the presence of T cells in the TME should be preserved to restore their antitumor potential. Thus, tailoring novel strategies that selectively target key points controlling the suppressed TME without affecting T cells is an interesting perspective, and in this regard, new drug-loaded nanosystems that selectively target tumor and tumor-promoting cells while saving T cells could be considered as a novel and effective approach in cancer therapy (41). Therefore, a detailed knowledge of the brain TME and immune evasion patterns is essential for the design of successful treatment strategies.

As regards the limitations of our study, metastatic brain biopsies are rare and it is difficult to collect freshly resected specimens in sufficient quantities for functional studies. Moreover, myeloid cells are very fragile and require a careful manipulation, and this contributes to the difficulty to perform these studies. Nevertheless, we were able to perform the analysis of myeloid cells’ immune suppressive activity and the study of cell proliferation in a single case of GBM and of a BrM. However, given the implication of these results, future efforts are required to confirm them. In addition, our study does not have a large enough sample size to conduct all the comparisons between the different tumor types in all the different experiments. Another issue concerns the prior treatments of metastatic patients, as opposed to GBM that are, instead, treatment-naïve. Therefore, future efforts should incorporate the present findings by addressing the impact of prior treatments on the composition of the TME, a factor we did not consider for this study. Nevertheless, we have previously demonstrated that, despite therapy, the TME of relapsing GBM maintains a similar infiltration pattern as the primary tumor (25, 42). This suggests that, regardless of the patient’s therapy, the recruitment of blood-derived cells with immune suppressive activity may also be a hallmark of BrM. In conclusion, our analysis reveals that, regardless of its origin, the presence of a brain tumor sculpts the microenvironment toward an immunosuppressed state, in which blood-derived cells with immunosuppressive activity and proliferative potential play a prominent role. Given the important role these cells play in sustaining tumor growth, it is crucial to understand the immunological component of the TME at both the tumor and systemic levels for the identification of targets and the development of effective therapeutic approaches.

## Data availability statement

The raw data supporting the conclusions of this article will be made available by the authors, without undue reservation.

## Ethics statement

The studies involving humans were approved by Veneto Institute of Oncology–IRCCS of Padova, Italy (MDSC_SNC 2016/13) and Padova and Florence University Hospitals (NOI_NCH 1536/19). The studies were conducted in accordance with the local legislation and institutional requirements. The participants provided their written informed consent to participate in this study.

## Author contributions

Conceptualization, SuM; methodology, SuM; formal analysis, BM; investigation, BM, MR, AT, SaM, GB, and LP; resources, CB and ADP; data curation, BM, MR, and SaM; writing, BM, CB, and SuM; supervision, SuM; project administration, SuM; funding acquisition, SuM. All authors contributed to the article and approved the submitted version.
