# Supplementary material for: The immune cell landscape of glioblastoma patients highlights a myeloid-enriched and immune suppressed microenvironment compared to metastatic brain tumors
Source: Front Immunol. 2023 Oct 23;14:1236824. doi: 10.3389/fimmu.2023.1236824 (PMC10626453; doi:10.3389/fimmu.2023.1236824)
Supplement: Supplementary file 1 [file DataSheet_1.docx]

*
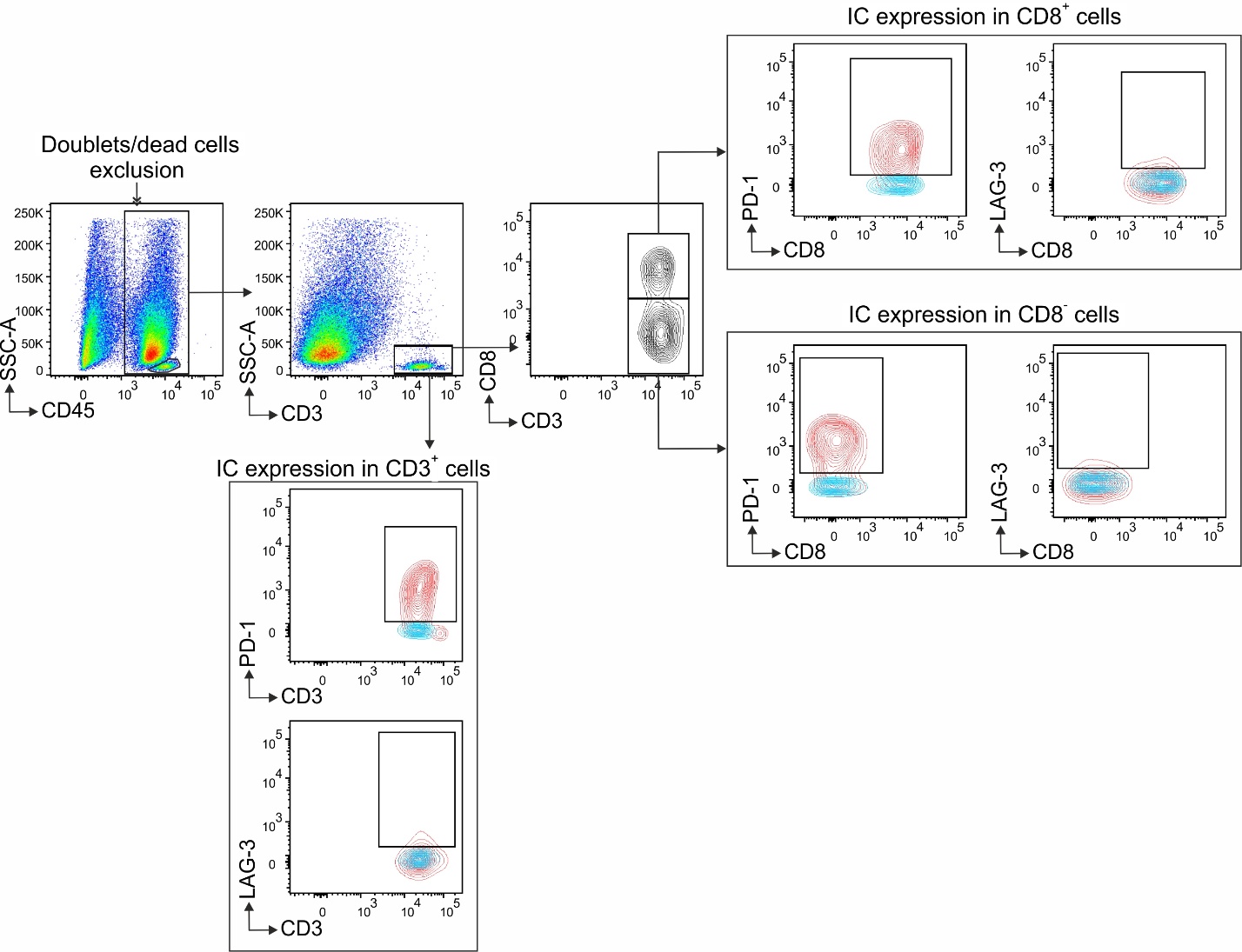
***SUPPLEMENTARY MATERIAL**

**Figure S1: T cell infiltrate and exhaustion markers in the TME of BrM and GBM.** Representative flow cytometry gating strategy for the identification of the different lymphoid subsets in tumor samples and their expression of exhaustion markers. After morphological evaluation and the exclusion of doublets and dead cells (not shown), leukocytes were identified on the basis of their CD45 expression. In addition, for the case in which total lymphocytes were considered, they were gated in live cells as SSC-A^low^/CD45^+^ cells. Then, CD3^+^ were selected among total CD45^+^ leukocytes and CD8^+^ and CD4^+^ cells were discriminated in CD3^+^ gate. Particularly, CD8^+^ cells were gated as CD3^+^/CD8^+^ (as shown) or CD3^+^/CD4^-^ cells when CD8 marker was not available, while CD4^+^ cells were identified as CD3^+^/CD8^-^ (as shown) or CD3^+^/CD4^+^ cells (not shown). PD-1, LAG-3 and Tigit (not shown in the example) were further evaluated in each of these populations basing on fluorescence minus one (FMO) controls (overlapped in blue).


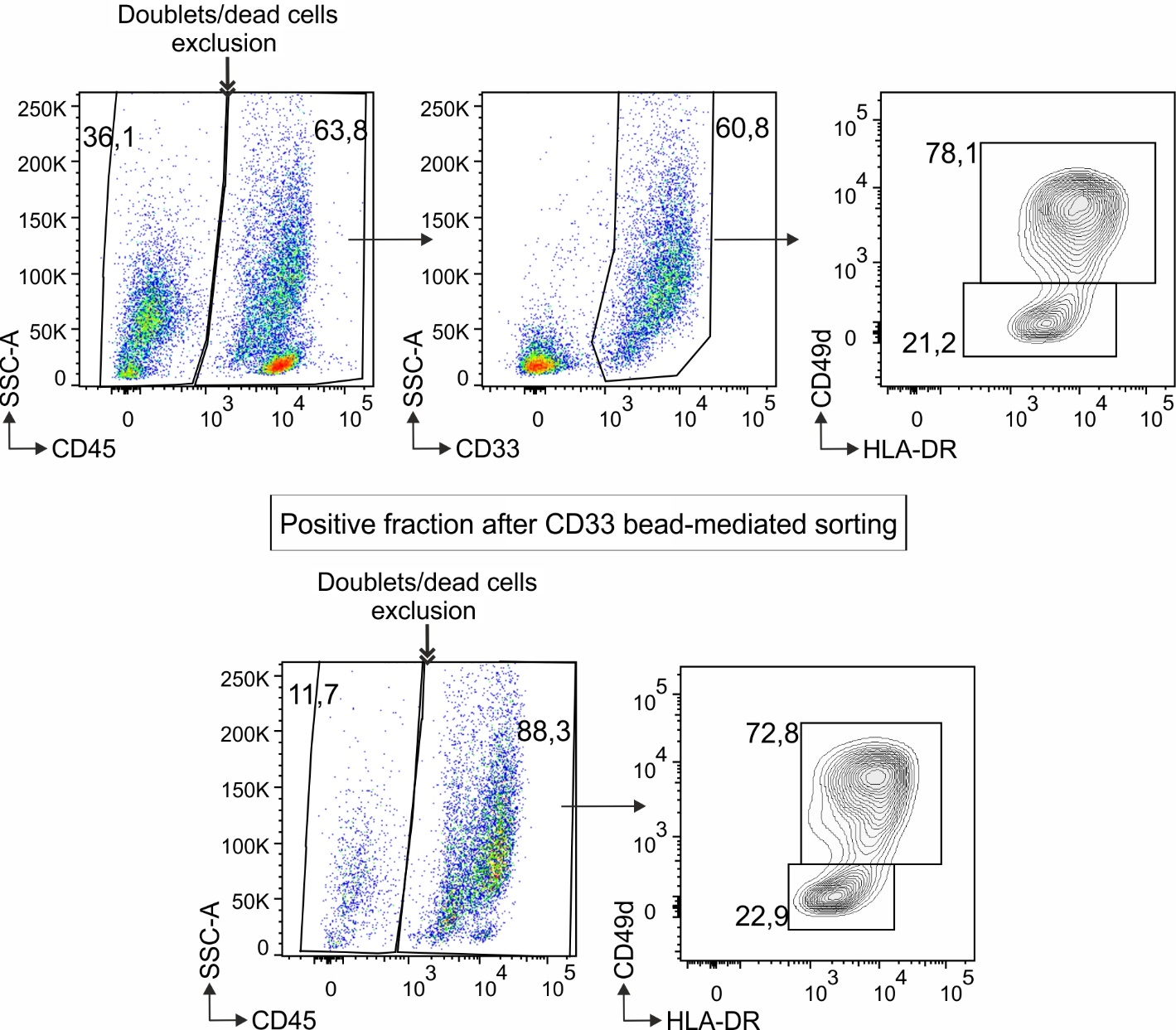


**Figure S2:** Representative flow cytometry gating strategy for the isolation of CD33^high^ cells from a lung BrM. After morphological evaluation and the exclusion of doublets and dead cells (not shown), leukocytes were identified on the basis of their CD45 expression. Macrophages were further discriminated in the CD45^+^ gate based on their CD33 expression. Finally, BMDM and MG were further differentiated in the CD33^high^ subset based on the combined expression of CD49d and HLA-DR, with BMDM distinguished as CD49d^+^/HLA-DR^+^ and MG as CD49d^-^/HLA-DR^+^, respectively. After immunomagnetic bead-based cell sorting, the sorted fraction were stained with the same markers except for CD33 to assess the purity of the sorting procedure.


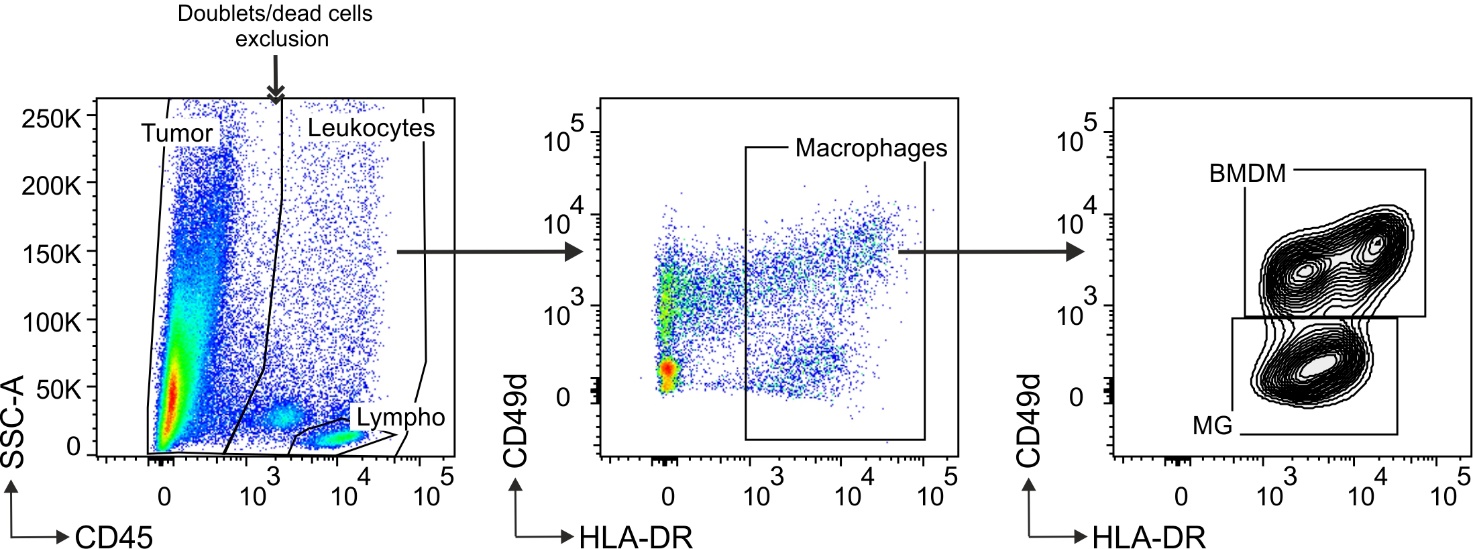
**Figure S3: Representative flow cytometry gating strategy for the identification of the different cell populations in tumor samples used for BrdU proliferation analysis.** After morphological evaluation and the exclusion of doublets and dead cells (not shown), leukocytes and tumor cells were identified on the basis of their CD45 expression. In addition, lymphocytes were gated among live cells as CD45^+^/SSC^low^ cells. Macrophages were further discriminated in the CD45^+^ gate based on the combined expression of CD49d and HLA-DR and further differentiated in the two populations of BMDM (CD49d^+^/HLA-DR^+^) and MG (CD49d^-^/HLA-DR^+^).
